# Non-coding RNAs and glioblastoma: Insight into their roles in metastasis

**DOI:** 10.1016/j.omto.2021.12.015

**Published:** 2021-12-22

**Authors:** Seyed Mojtaba Mousavi, Maryam Derakhshan, Fatereh Baharloii, Fatemeh Dashti, Seyed Mohammad Ali Mirazimi, Maryam Mahjoubin-Tehran, Saereh Hosseindoost, Pouya Goleij, Neda Rahimian, Michael R. Hamblin, Hamed Mirzaei

**Affiliations:** 1 Department of Neurosciences and Addiction Studies, School of Advanced Technologies in Medicine, Tehran University of Medical Sciences, Tehran, Iran; 2 Department of Pathology, Isfahan University of Medical Sciences, Isfahan, Iran; 3 Department of Cardiology, Chamran Cardiovascular Research Education Hospital, Isfahan University of Medical Sciences, Isfahan, Iran; 4 School of Medicine, Kashan University of Medical Sciences, Kashan, Iran; 5 Student Research Committee, Kashan University of Medical Sciences, Kashan, Iran; 6 Department of Medical Biotechnology and Nanotechnology, Faculty of Medicine, Mashhad University of Medical Sciences, Mashhad, Iran; 7 Brain and Spinal Cord Research Center, Imam Khomeini Hospital, Tehran University of Medical Sciences, Tehran, Iran; 8 Department of Genetics, Faculty of Biology, Sana Institute of Higher Education, Sari, Iran; 9 Endocrine Research Center, Institute of Endocrinology and Metabolism, Iran University of Medical Sciences (IUMS), Tehran, Iran; 10 Laser Research Centre, Faculty of Health Science, University of Johannesburg, Doornfontein 2028, South Africa; 11 Radiation Biology Research Center, Iran University of Medical Sciences, Tehran, Iran; 12 Research Center for Biochemistry and Nutrition in Metabolic Diseases, Institute for Basic Sciences, Kashan University of Medical Sciences, Kashan, Iran; 13 Department of Internal Medicine, Firoozgar Hospital, School of Medicine, Iran University of Medical Sciences, Tehran, Iran

**Keywords:** glioblastoma, non-coding RNA, metastasis, microRNA, long non-coding RNA, circular RNA

## Abstract

Glioma, also known as glioblastoma multiforme (GBM), is the most prevalent and most lethal primary brain tumor in adults. Gliomas are highly invasive tumors with the highest death rate among all primary brain malignancies. Metastasis occurs as the tumor cells spread from the site of origin to another site in the brain. Metastasis is a multifactorial process, which depends on alterations in metabolism, genetic mutations, and the cancer microenvironment. During recent years, the scientific study of non-coding RNAs (ncRNAs) has led to new insight into the molecular mechanisms involved in glioma. Many studies have reported that ncRNAs play major roles in many biological procedures connected with the development and progression of glioma**.** Long ncRNAs (lncRNAs), microRNAs (miRNAs), and circular RNAs (circRNAs) are all types of ncRNAs, which are commonly dysregulated in GBM. Dysregulation of ncRNAs can facilitate the invasion and metastasis of glioma. The present review highlights some ncRNAs that have been associated with metastasis in GBM. miRNAs, circRNAs, and lncRNAs are discussed in detail with respect to their relevant signaling pathways involved in metastasis.

## Graphical abstract

**Figure:**
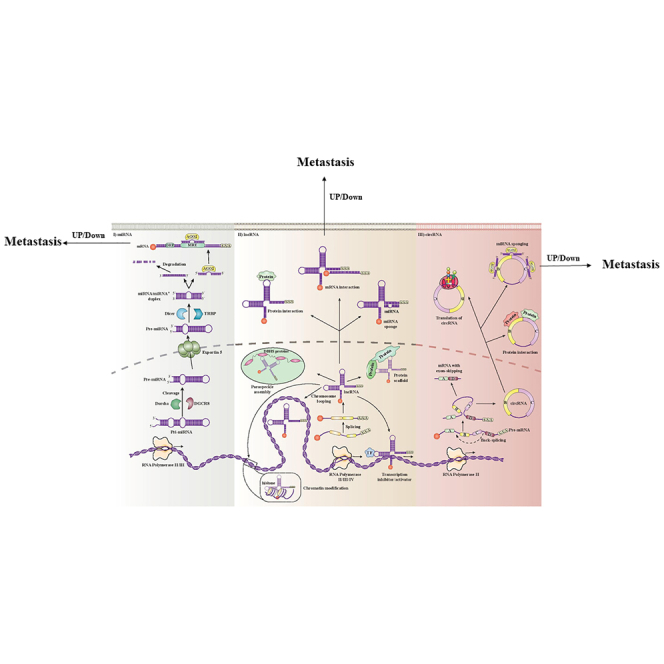

## Teaser

These recent advances will shed more light on a thorough understanding of ncRNA function and the possible underlying molecular mechanisms involved in the regulation of metastasis. Their successful translation will depend on further interdisciplinary collaboration to improve tolerance, specificity, and delivery.

## Introduction

Glioma is one of the most common primary tumors in the central nervous system (CNS) (∼30%) and is the most aggressive and lethal. Glioma is further histologically divided into five distinct subtypes: oligodendroglioma; astrocytoma; medulloblastoma; ependymoma; and glioblastoma multiforme (GBM).^1^^,^^2^ Despite recent development in treatment options for glioma, including various chemotherapy regimens, advanced radiotherapy, and surgical resection techniques, patients with glioma often have a poor prognosis.^3^ Accurate classification of the tumor and categorization of patients require a comprehensive understanding of the tumor properties and biology. During the last several decades, many studies have been carried out on gliomas to discover novel molecular biomarkers through the identification of abnormal gene expression, epigenetic alterations, and genetic mutations.^4^

GBM or grade IV glioma is the most common type of brain cancer. GBM is one of the deadliest malignant tumors, with a very short life expectancy. The 5-year survival of patients with GBM is only about 5%. Up to 60 Gy of radiotherapy delivered over 3 weeks accompanied by daily administration of temozolomide (TMZ), followed by an additional six cycles of adjuvant TMZ, is the standard therapy for patients with a sufficient performance status.^5^ Recent clinical trials have demonstrated improved survival with the administration of electric field therapy, called “tumor-treating fields.” The survival of glioblastoma patients could be prolonged by approximately 5 months by the addition of electric field therapy to the standard therapy.^6^

Abnormal regulation of proliferation and inhibition of apoptosis are vital processes in tumorigenesis that ultimately lead to the development of tumors. Metastasis is accepted to be one of the hallmark features of cancer.^7^ A major obstacle for the treatment of metastatic tumors is the biologic heterogeneity of the tumor cells. This biologic heterogeneity also contributes to the late diagnosis of many cancers. Oncogenes are tumor-promoter genes, which often control cellular proliferation and apoptosis. Scientists have recently focused on discovering specific biomarkers that can be related to genes that encode protein production in cancer.^8^ The human genome can be divided into two broad categories of sequences: (1) a minor group of sequences that are protein-coding genes, accounting for perhaps 20,000 genes or 2% of the entire genome and (2) a major group of sequences that encode non-coding RNA molecules, which are not translated into proteins. Non-coding RNAs (ncRNAs) regulate the translation of other RNAs and control the production of functional proteins from protein-coding transcripts.^9^ Many studies have shown the diagnostic or prognostic potential of ncRNAs.^10^, ^11^, ^12^, ^13^

Long ncRNAs (lncRNAs) are about 200 nt in length and are mostly polyadenylated transcripts. These transcripts are usually translated by RNA polymerase II and can be regulated by many different transcription factors. Among all the various ncRNAs, lncRNAs account for the largest fraction (>80%) and have greater variability compared with microRNAs (miRNAs).^14^ lncRNAs have been found to play a crucial role in the conversion of mRNAs into small interfering RNAs. miRNAs have been recently shown to control lncRNAs, and conversely, lncRNAs are able to regulate the expression of miRNAs, thus forming a two-way regulatory network with major roles in many cellular pathophysiological processes.^15^ This cross-talk between miRNAs and lncRNAs means that miRNA can induce destruction of lncRNAs, while lncRNAs can alter the levels of miRNAs and regulate their function, such as through sponging the miRNAs.^16^ Moreover, lncRNAs can compete with miRNAs for binding to mRNAs, while miRNAs can be produced from lncRNAs.^17^ This cross-regulatory interaction between miRNAs and lncRNAs and its abnormal expression have potential diagnostic and therapeutic implications in many cancers, including glioma.^18^, ^19^, ^20^ ncRNAs, such as lncRNAs and miRNAs, may be used as prognostic and predictive biomarkers in glioma patients, as their effects on gene expression can affect glioma progression and metastasis.

Circular RNAs (circRNAs) are a type of ncRNA that has only recently been discovered and are highly conserved across different species. circRNA expression patterns are tissue specific and tumor-stage dependent.^21^, ^22^, ^23^ circRNAs may have different functions, including sponging miRNAs, regulating gene transcription, and binding to proteins, and some circRNAs can be translated to proteins. These encoded peptides may serve as a new class of drug targets.^24^^,^^25^ circRNAs are frequently found in neural tissue and are expressed differentially in different parts of the brain. This finding may be due to the fact that there are a variety of different protein-coding genes, which produce different circRNAs, splicing factors, and RNA-binding proteins (RBPs), which regulate the formation of circRNAs.^26^

Piwi-interacting RNAs (piRNAs) are another type of ncRNA that are 26–30 nt in length and bind to Piwi proteins. These short RNAs were originally discovered in germline cells and are considered to be key regulators for germline maintenance.^27^ A growing body of evidence has now extended our knowledge of the biological significance of piRNAs, because they can also regulate gene expression in somatic cells through transposon silencing, epigenetic programming, DNA rearrangement, mRNA turnover, and translational control. Accumulating evidence has revealed that the dysregulation of piRNAs may cause epigenetic changes and contribute to many diseases.^28^

Herein, we highlight the role of some ncRNAs that have been associated with metastasis in GBM. miRNAs, circRNAs, and lncRNAs will be discussed in detail. Due to the high importance of RNAs in gliomas, many studies have been conducted on ncRNAs; however, in the present study, the effects of ncRNAs on metastasis-related genes and pathways are discussed, which distinguishes this review from others.

## Metastasis and glioma

Glioma stem cells (GSCs) with a low proliferation rate are found in hypoxic areas of GBM tumors, such as periarteriolar pits.^29^^,^^30^ GSCs express angiomotin, thrombospondin, and ephrin (EphA5), which inhibit the formation of new blood vessels.^31^ GSC niches and normal hematopoietic stem cell (HSC) niches are similar in that both are located in periarteriolar regions, are hypoxic, and consist of similar functional proteins that attract biomolecules and receptors that bind to the stem cells.^32^ It has been shown that the mesenchymal stem cells (MSCs) present in GSC niches promote a proliferative and regenerative environment, which can stimulate GSCs *in vitro*, possibly through the interleukin-6 (IL-6)/STAT3 signaling pathway, with the resultant induction of glioma-associated human MSCs (GA-hMSCs) *in vivo*.^33^ CD105-positive MSCs are located in the periarteriolar space and express abundant amounts of SDF-1α and OPN. These two molecules are chemoattractant receptors that attract CD44^+^ and CXCR4^+^ GSCs to the periarteriolar niche; therefore, GSCs are protected from chemotherapy and remain in an inactive state.

GSCs in the quiescent state require the production of angiogenic factors to switch into a more proliferative and metastatic phenotype. This switch to a more aggressive and proliferative state has been demonstrated in many studies, particularly in patients with recurrent GBM,^34^ possibly through the proneural-mesenchymal transition (PMT). This shift was reported to be induced by microenvironmental cues, including those arising from stromal cells.^34^^,^^35^ Irradiation can also cause the PMT, which is followed by increased expression of CD44 and YKL-40 (chitinase-3-like protein 1), as well as nuclear factor κB (NF-κB), STAT3, and CEBPB, which are master transcription factors in PMT.^34^^,^^36^, ^37^, ^38^ This shift was reported to be associated with enhanced activity of the YAP/TAZ signaling axis and increased colony formation and resistance in GSCs.^34^ This assumption was in agreement with a study by Elena et al. indicating that the aggressive mesenchymal phenotype in GBM was associated with over-expression of the YKL-40 marker, which led to extracranial metastasis. In their study, extracranial metastasis appeared at a median 8.5 months after GBM was initially diagnosed, with an estimated survival time of 12 months.^39^ When Sullivan et al. compared primary GBM cells to GBM circulating tumor cells (CTCs) isolated from both patients and mouse-patient-derived xenograft (PDX) models, they found that MES markers were more abundant than N/PN markers in the metastatic cells (CTCs).^40^

The use of RNA *in situ* hybridization in primary GBM tumor cells revealed a subgroup of mesenchymal cells with high migratory properties. Metastatic lesions predominantly showed a mesenchymal phenotype, along with extra mutations in a small group of patients with systemic spread of GBM. It seems that the niche of the metastatic sites governs the progression of metastasis. After this initial spread, an angiogenic transition would be able to activate the quiescent cells,^31^^,^^41^ leading to their removal from the niche.^30^^,^^42^ This leads to more rapid proliferation and colonization of the GSCs in distant organs. In order to achieve an extracranial metastasis, the degradome (expression profile of proteases) of the metastatic GSCs has to adapt itself to the new microenvironment in distant organs.

**Figure 1:**
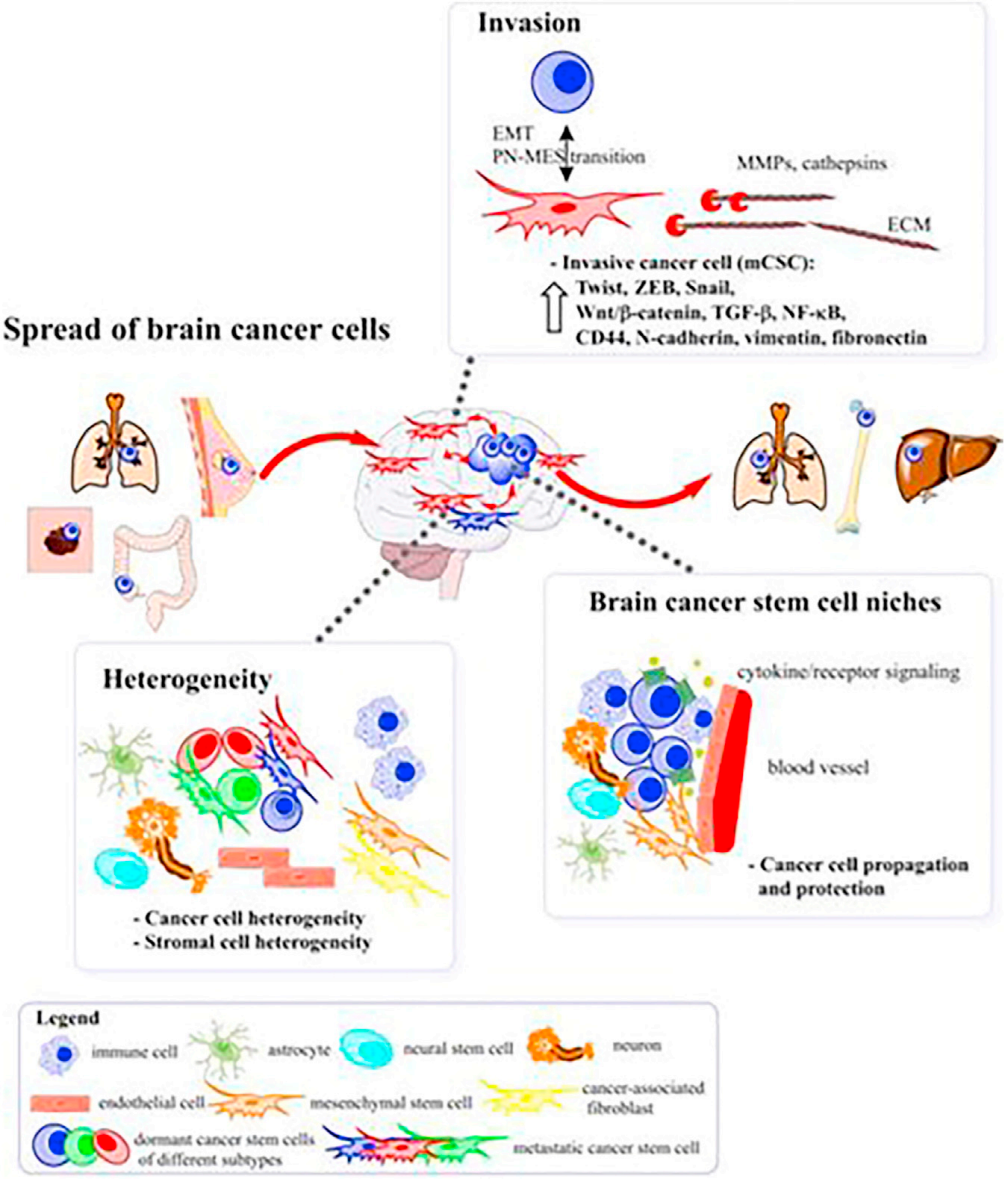
Possible routes of spread of malignant cells in GBM The microenvironment of brain cancer involves various subtypes of tumor cells in addition to different subtypes of stromal cells, which are physiologically located in the brain (neurons, astrocytes, and microglia) or invade the brain during the progression of the tumor (fibroblasts, mesenchymal stem cells, lymphocytes, and macrophages). Dormant cancer stem cells (dCSCs) within a number of tumors (colorectal, lung, breast, and melanoma) could initially transform into metastatic cancer stem cells (mCSCs) to develop a niche by colonizing the brain tissue. On the other hand, dormant glioblastoma stem cells (dGSCs) are present in their conserved niche in the brain; however, exogenous stimuli, such as chemotherapy, radiotherapy, hypoxia, or alterations in the endogenous microenvironment, may enhance the proliferation and invasion of these malignant cells. The PMT with the resultant transformation of dGSCs into mGSCs is triggered by cytokine production and signaling.

It can be concluded that, although still not completely identified, the metastatic niche may be considered as a novel therapeutic target in GBM patients with distant metastasis (Figure 1). Zhao et al. described some examples of niche-targeting therapeutic methods against GSCs that have been tested using complex *in vivo* models and organoid cultures and might be applicable in the clinical setting.^43^ This approach could be used as an adjuvant therapy in combination with radiotherapy or surgical resection and could inhibit the development of recurrence and metastasis.

Extracranial metastasis is rare, occurring in up to 0.5% of patients with GBM. Although GBM tumors are locally invasive, they rarely spread beyond the brain.^44^ However, the rapidly progressive and destructive nature of the tumor in the brain results in an unfavorable prognosis and earlier death compared with systemic metastasis of other cancers. Extracranial metastasis is usually not detected at autopsies, as the underlying cause of death is already known.^44^ Therefore, two important questions arise: the first is whether spontaneous metastasis in GBM patients is actually as rare as supposed, and the second is, if so, which of the phases of metastasis prevents the development of spread to other organs? Metastasis may occur in GBM patients undergoing craniotomy and surgical resection as GBM and/or GSC cells may be released into blood vessels during surgery. This notion was initially suggested by the presence of GBM metastases in skin scars and soft tissue in proximity to the original site of the craniotomy,^44^ as well as presence of extracranial metastases located within ventriculo-peritoneal shunts.^45^^,^^46^ However, the spreading of GBM cells beyond the brain has not been clearly established by laboratory evidence.

Possible barriers to metastasis in GBM patients are (1) presence of the blood-brain barrier (BBB), providing protection; (2) lack of lymphatic vessels located in the CNS preventing lymphatic metastasis; (3) inhibition of growth of GBM cells outside the brain caused by the immune system; and (4) possible inability of GBM cells to invade or destroy the extracellular matrix (ECM) in organs other than the brain.

Current experimental studies suggest that the BBB is unable to effectively prevent penetration of cells through it to reach the extracranial space.^47^ Moreover, intercellular interactions between differentiated MSCs (pericytes), cancer associated fibroblasts (CAFs), and even tumor cells themselves may disrupt the blood vessels in GBM tissue.^47^ In the CNS of mice and humans, functioning lymphatic vessels were found to be present, which are covered by typical endothelial cells in dural venous sinuses with subsequent involvement of deep cervical lymph nodes.^48^ The idea that lymph node metastasis may occur in GBM patients prior to any surgical resection has been confirmed by previous studies.^49^ Lymphatic metastases account for approximately 50% of GBM metastasis, followed by pleural/lung (50%), bone (31%), liver (12%), and skin, respectively.^44^ In addition to lymphatic vessels, GBM and GSC metastasis can occur by invasion into the cerebrospinal fluid (CSF). CSF acts physiologically as a “cushion” for the CNS as it maintains the ionic balance of the extracellular space and contains proteins with specific functions, including insulin-like growth factor 1 (IGF1) and 2 (IGF2) and Sonic hedgehog, which contribute to maintenance of neural stem cell properties in their niches located in the subventricular space.^50^ These factors are also up-regulated in GSCs in some GBM patients.^51^ They can also condition metastatic GSCs (mGSCs), causing neural stem cell properties to be preserved in their subventricular zone niche.^50^ Hematogenous metastasis occurs when GSCs reach the systemic blood circulation via the CSF and lymphatic vessels. In a study by Müller et al., CTCs were found in the systemic blood circulation in approximately 21% of patients with GBM, as shown by a specific neural biomarker glial fibrillary acidic protein (GFAP), in conjunction with mutations or over-expression of the EGFR gene. This suggests that EGFR gene mutation is important for GBM hematogenous metastasis.^52^ Levels of CTCs were generally not elevated after surgical resection. Metastases that do not lead to any clinical symptoms are often ignored, despite the unfavorable overall survival of GBM patients. However, evaluation of CTCs may be beneficial in GBM patients with a long life expectancy (see sections below).

GSCs are able to protect themselves from the immune system within the systemic blood circulation,^53^ because they can stimulate the production of myeloid-derived suppressor cells (MDSCs), contributing to the ability of GSCs to evade natural killer (NK) cells.^54^^,^^55^ Apparently, GSCs lack Toll-like receptors on their surface, which contributes to avoidance from attack by the immune system.^56^ In patients with a normal immune system, the majority of circulating tumor cells are identified and killed by NK cells.^57^ However, immuno-deficient patients, including those undergoing chemotherapy or radiotherapy, have a higher likelihood to suffer disseminated metastases. This suggests a correlation between the competence of the immune response and metastasis risk in GBM patients.

There are several studies that have examined survival and metastasis in GBM patients, with different numbers of patients and ages at the time of diagnosis. Lun et al. carried out a study on 88 patients and found that the median overall survival of GBM patients was only approximately 10 months after diagnosis and about 8.5 months from the diagnosis of metastasis.^58^ Among different metastatic sites examined, metastasis to the lung was associated with the worst prognosis. Intensive therapy, including the application of shunts for the CSF, increased the overall survival of patients with metastases. This finding questioned the assumption that treatment of GBM patients may enhance the chance of metastasis and result in a worse prognosis. A meta-analysis by Pietschmann et al. on 150 patients found a median survival of about 6 months from detection of metastasis.^59^ A cohort study compared 84 patients with GBM metastasis with patients without metastasis and found that younger patients with GBM metastasis had a longer overall survival, presumably due to the presence of an effective immune response. Notably, resection of the tumor increased the time interval to occurrence of metastasis outside the brain compared with biopsy alone. Surgical excision of the tumor combined with chemotherapy or radiotherapy may be associated with an even longer interval before occurrence of metastasis and better overall survival. This notion was corroborated by a meta-analysis on 115 younger GBM patients, which found that the time interval between detection of metastasis and death was longer when patients had surgical excision of the mass or received chemotherapy or radiotherapy.^60^ In another study, metastasis to the liver was associated with the worst outcome and shortest overall survival. However, treatment with bevacizumab, an anti-vascular endothelial growth factor (VEGF) monoclonal antibody, may paradoxically enhance progression of the disease through hypoxia induction, which may be associated with earlier metastasis.^61^

In conclusion, these clinical studies suggest that GBM cells may spread beyond the brain via CSF and lymphatic vessels, finally reaching the bloodstream or other lymph nodes. Notably, hematogenous metastases do not appear to be encouraged by surgical excision of the tumor and may even be associated with a better prognosis because they stimulate the immune response.

## Non-coding RNAs and glioma

The uncontrolled proliferation, invasion, and migration of cancer cells are considered to be the hallmark features of tumors. The survival of cancer patients is affected by the migration of the malignant cells and their ability to invade into other organs.^62^ Specific interactions between tumor cells and other normal cells, as well as the ECM, in combination with other physiological processes results in the active movement of cells.^63^ The epithelial-mesenchymal transition (EMT) is initiated through alteration of transcription factors (Snail, ZEB, and Twist) and secretion of various growth factors, including fibroblast growth factor (FGF)-2, transforming growth factor β (TGF-β), platelet-derived growth factor (PDGF), and VEGF. Tumor cells also produce different types of proteases that facilitate the invasion of malignant cells into healthy brain tissue by degrading the ECM.^64^ Furthermore, glioblastomas are associated with disturbances in apoptosis, including increased levels of anti-apoptotic proteins, such as phosphatidylinositol-4,5-bisphosphate 3-kinase (PI3K) and the anti-apoptotic family protein B-cell lymphoma 2 (Bcl-2).^65^ The regulatory mechanisms that control the cell cycle via retinoblastoma (RB) and p53 proteins are not active, leading to uncontrolled proliferation and tumor progression.^66^ Loss of cellular tissue integrity, dysregulated cell death, and uncontrolled proliferation are all hallmarks of cancer development. ncRNAs play a crucial role in regulating these cellular characteristics in cancer patients.^67^

**Figure 2:**
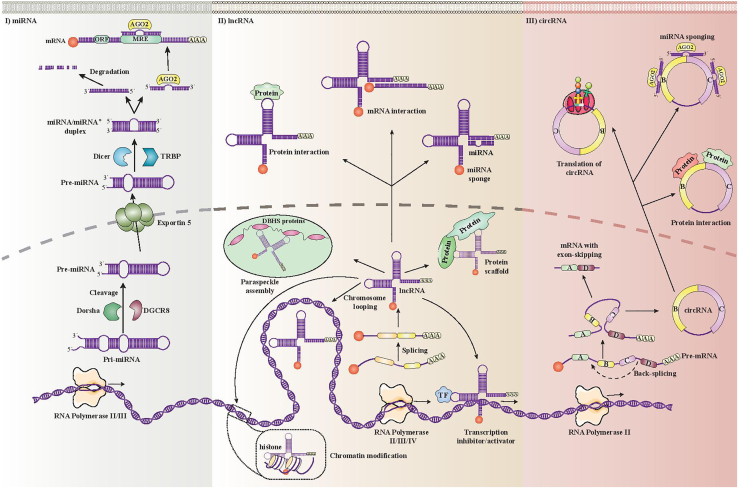
Biogenesis and function of ncRNAs miRNAs, lncRNAs, and circRNAs. (I) RNA polymerase II/III transcribes miRNAs initially as primary miRNAs (pri-miRNAs). pri-miRNAs are composed of a stem-loop structure. DiGeorge syndrome critical region 8 (DGCR8) and Drosha cut the stem of pri-miRNA in the nucleoplasm, which results in production of precursor miRNA (premiRNA). Exportin-5 contributes to the removal of pre-miRNAs from the nucleus. The pre-miRNA is further processed in the cytoplasm through the activity of transactivation response element RNA-binding protein (TRBP) and Dicer. A duplex composed of two miRNAs (miRNA/miRNA∗) is created after pre-miRNA loop is cleaved. The strand attaches to Argonaute 2 (AGO2), which is the major protein of the RNA-induced silencing complex (RISC). miRNAs are included in the RISC. They can regulate gene expression in a posttranscriptional manner through formation of miRNA response elements (MREs) by binding to specific sites located on the 3′ untranslated region (3′ UTR) of mRNA. (II) lncRNAs have a similar biogenesis to mRNAs, in that a number of lncRNA transcripts are polyadenylated at 3′ and 5′ ends, coated, and spliced. (III) circRNAs can be transcribed from protein-coding genes that contain introns or exons. “Back-splicing” can be triggered by various mechanisms, including dimerization of proteins, pairing of introns, or lariat intron formation.

There is an expanding number of lncRNAs that have been found to be highly expressed in GBM patients, which highlights their important role in proliferation and development of cancer (Figure 2). miRNAs have been studied for a long time, and a substantial amount of data about their target genes and signaling pathways now exists. There still remain some missing gaps about how miRNAs interact with lncRNAs, even after lncRNAs were discovered. An association between miRNAs and lncRNAs has not been found in all cases. The current knowledge about the interaction of miRNAs and their target genes with lncRNAs and signaling pathways with relevance to glioma is summarized in Figure 3. lncRNAs, miRNAs, and their target genes particularly affect the signaling pathways, Wnt/β catenin, Notch, and PI3K/Akt/mTOR. A number of lncRNAs and miRNAs have the ability to control multiple different signaling pathways. Although these ncRNAs have a complex network, they may act as novel predictive or prognostic biomarkers in patients with cancer (Figure 3).

Nevertheless, other types of ncRNAs that have been less well investigated may function as biomarkers in glioma patients. These ncRNAs include circRNAs, small nucleolar RNAs (snoRNAs), small Cajal body-specific RNAs (scaRNAs), and piRNAs. snoRNAs have many biological functions, such as structural alteration of other RNAs, miRNA precursors, assembly, and activation of telomerase enzymes.^68^ A genome-wide analysis study of small ncRNAs in pediatric glioma patients found that 118 separate members were over-expressed (72 CD-Box, 26 HACA-Box, 3 scaRNAs, and 17 snoRNAs) and 39 separate members were under-expressed (one snoRNA and 38 CD-Box).^69^ The snoRNA SNORD was found to control the EMT and to affect the response to therapy with temozolomide.^70^ SNORD76 (C/D box snoRNA U76) was inversely associated with HOTAIR expression and could affect the proliferation of cells *in vivo* and *in vitro*. The up-regulated SNORD76 level resulted in a decreased expression of p107, *cyclin A1*, and *cyclin B1* genes and an increased expression of *Rb* gene.^71^ High expression of snoRNA SNHG18 may lead to radiotherapy resistance of glioma cells by inhibiting Semaphorin 5A.^72^ There is no evidence yet regarding the expression pattern of SNHG18 in glioma tissue. The snoRNA SNHG1 was found to be up-regulated in glioma cells and was associated with enhanced proliferation and decreased apoptosis of tumor cells. However, the precise cellular mechanisms and biological targets remain to be elucidated.^73^ scaRNAs play biological roles in glioma; however, their role remains poorly understood. In addition, scaRNAs may play an important role in the pathogenesis of other malignancies.^74^^,^^75^ circRNAs are a type of ncRNA that may function as specific sponges of miRNAs.^76^^,^^77^ A group of circRNAs has been recently shown to have a crucial role in the development of glioma. circ-TTBK2 is over-expressed in glioma patients and *in vitro* in U87 and U251 cell lines. The under-expression of circ-TTBK2 along with up-regulation of miR-217 resulted in inhibition of cancer growth *in vivo*.^78^ Moreover, circFBXW7 was under-expressed in glioma tissue in comparison to normal adjacent brain tissue. In addition, up-regulated levels of circFBXW7 correlated with a more favorable overall survival in glioma patients.^79^ circZNF292 is another ncRNA that promotes the formation of new blood vessels. Moreover, this circRNA can stimulate cell cycle progression and proliferation acting via the Wnt/β-catenin signaling pathway and was up-regulated in U251 and U87MG cell lines.^80^ Unfortunately, the expression level of circZNF292 in glioma patients has not been clearly identified.^81^ piRNAs are a group of small ncRNAs that is produced from RNA precursors different from the precursors of miRNAs.^82^ piRNAs contribute to regulation of gene expression through inducing alterations at the epigenetic level,^83^ leading to destruction of mRNAs.^84^ PIWI protein and piRNAs form a complex that controls differentiation, proliferation, and apoptosis of cells.^85^, ^86^, ^87^ Furthermore, piRNAs can regulate other biological processes through interaction with siRNAs and miRNAs.^88^^,^^89^ The PIWIL1/MEG3/miR-330/RUNX3 signaling axis controls the blood-tumor barrier, demonstrating the significance of ncRNAs in cellular processes.^90^

## Biogenesis of non-coding RNAs

miRNAs are the group of ncRNAs that have been most well studied. The initial phase of miRNA formation involves a structure composed of two stem loops (known as pri-miRNA). This two-stem-loop pri-mRNA is cleaved into a single-stem-loop structure (pre-miRNA) by the activity of the complex between DGCR8 and Drosha. This pre-miRNA is further cleaved by Dicer to a double-stranded structure (miRNA). This miRNA is added to a member of the protein family called Argonaute, which leads to formation of the miRNA-induced silencing complex (miRISC) (Figure 2). In addition to the miRISC, miRNAs are able to modulate posttranscriptional expression of genes via a mechanism mediated by another Argonaute family protein, which results in fragmentation and suppression of mRNA sequences (Figure 2).^91^

RNA polymerase II enzyme contributes to the production of lncRNAs through transcription of genes located in exonic, intergenic, or distal protein-producing sequences of the human genome. This pre-mature lncRNA becomes polyadenylated at the 3′ end and attached to methyl-guanosine at the 5′ end.^92^ This structure is often subjected to alternative splicing to form different proteins.^93^ Alternative splicing can be categorized into three pathways. Initially, lncRNAs undergo interactions with different splicing factors, which leads to the production of RNA-RNA complexes along with molecules of pre-mRNA. They can finally regulate the remodeling of chromatin; thereby, mixing of the target genes is achieved.^94^

Traditionally, the functions of lncRNAs have been classified either as *cis* (when the target gene is located adjacent to the lncRNA sequence) or *trans* (when the target gene is located far away from the lncRNA sequence). *cis* lncRNAs typically stimulate or suppress transcription of adjacent genes by mechanisms such as triggering various epigenetic alterations in chromatin, modifying the DNA structure (looping of chromosomes), and interacting with different transcription factors. The *trans* function of lncRNAs mainly takes place in the nucleus, such as assembling the paraspeckles or interacting with different proteins in the nucleus. Various lncRNAs are able to leave the nucleus and sequester miRNAs within the cytoplasm, which leads to miRNA inhibition. Moreover, they can increase or decrease the half-life of proteins in the cytoplasm by interacting with them or cause stimulation or inhibition of mRNAs.

circRNAs are derived from pre-mRNA, and they are produced by the activity of group I or II ribozyme catalysts or splicing of spliceosomes.^95^ Spliceosomes may contribute to circRNA biogenesis, because both circRNAs and linear transcripts were decreased after suppression of canonical spliceosomes.^96^ circRNAs can arise from intronic, 3′ UTRs, 5′ UTRs, and also intergenic regions; however, they are predominantly transcribed from exons that encode proteins. Canonical and non-canonical cleavage processes contribute to the formation of circRNAs. Different from the orthodox splicing of cognate linear mRNAs, circRNAs can originate from a single gene locus through selection of alternative back-splicing sites, which are available in the CIRCpedia database.^97^

Up to now, circRNAs have been classified into three different types: intronic RNAs (ciRNAs); exonic circRNAs (ecircRNAs);^98^ and exon-intron circRNAs (ElciRNAs). One study suggested that ecircRNAs comprise the majority of circRNAs in plants and animals, and about 83% of them share sequences with genes encoding proteins.^99^ A number of ecircRNAs interact with RBPs and/or miRNAs. In addition, many ecircRNAs encircle another exon containing a canonical translation start codon.^100^

## MicroRNAs and metastasis in glioma

miR-376a-3p has been identified as an important miRNA involved in several malignancies.^101^^,^^102^ A high concentration of miR-376a-3p in the nucleus or cytoplasm was associated with increased tumor aggressiveness and could be used as a prognostic marker for glioma patients.^101^^,^^102^ According to bioinformatics, KLF15 is the downstream gene that binds to miR-376a-3p. Krüppel-like transcription factors (KLFs) are a family of transcription factors with a structure containing a C2H2 zinc finger domain. They are widely distributed in eukaryotes, comprising 9 unique proteins forming 18 KLF members.^103^^,^^104^ The hematopoietic, respiratory, and immune systems are all controlled by KLF15. Furthermore, KLF15 can influence tumor development by modulating the expression of downstream genes.^105^

Chen et al. identified the role of miR-376a-3p in modulating the invasiveness and migration of glioma cells and suggested potential mechanisms.^106^ Levels of miR-376a-3p were measured in glioma samples from 39 patients. The clinical histories of the patients were collected, and the relationship between the clinical presentation and the levels of miR-376a-3p was investigated. Then, the effects of miR-376a-3p on regulating the metastatic and proliferative properties of T98-G and U251 cell lines were investigated. Bioinformatics analysis was used to detect genes that could interact with miR-376a-3p. KLF15 was reported to be involved in the growth of glioma, and this was controlled by miR-376a-3p. miR-376a-3p was found to be down-regulated in glioma cells, and low levels were associated with higher metastatic capacity and a poor survival in glioma patients. In addition, higher levels of miR-376a-3p inhibited metastasis and proliferation in glioma tissue. KLF15 is a gene that can bind to miR-376a-3p and was inversely correlated with miR-376a-3p. KLF15 was up-regulated in glioma patients. *In vitro*, exogenous KLF15 had the ability to block miR-376a-3p effects on regulating glioma cell properties. Moreover, miR-376a-3p was associated with hematogenous metastasis via lymph nodes and distant metastasis, which was inversely correlated with KLF15.^106^

miR-623 expression has been shown to be down-regulated in many malignancies, including lung and stomach cancer. This miRNA has been shown to act as a tumor-suppressor gene.^107^^,^^108^ Earlier studies showed that mimics of miR-623 could reduce proliferation, migration, invasion, and colony formation of glioma cells. The volume of the tumor in an intracranial xenograft model in mice was significantly reduced when a treatment was applied that could up-regulate miR-940, suggesting that miR-940 may be proposed as glioma therapy.^109^^,^^110^ In addition, there is an ever-expanding list of miRNAs that have been associated with metastasis in GBM patients as summarized in Table 1.

**Table 1:** Metastasis-related microRNAs in GBM

miRNA	Expression status (up/down)	Target	Model (*in vitro*, *in vivo*, human)	Cell line	Ref
miR-376a-3p	down	KLF15	*in vitro*, human	U251, T98G	Chen et al.^106^
miR-4530	down	RTEL1	*in vitro*, human,	U251, T98G	Wang et al.^111^
miR-623	up	TRIM44	*in vitro*, *in vivo*	LN229, U251MG	Cui et al.^112^
miR-450a-5p	down	EGFR	*in vitro*	A172, SHG-44	Liu et al.^113^
miR-32	down	EZH2	*in vitro*, human	U87, U251, A172, U118	Peng et al.^114^
miR-767-5p	down	SUZ12	*in vitro*, human	T98, A172, U87, LN229, U251 U118	Zhang et al.^115^
miRNA-320c	down	cyclin D1, CDK6, MMP2, MMP9, N-cadherin, integrin β1	human		Lv et al.^116^
miR-3653	down		human		Chen et al.^117^
miR-93-5p	down	MMP2	*in vitro*, human	U87-MG	Wu et al.^118^
miR-375	down	RWDD3	*in vitro*, human	U251, U87	Ji et al.^119^
miR-140	down	ADAM9	*in vitro*, human	U87, U251, U373, U118, A172, LN18	Liu et al.^120^
miR-187	down	SMAD1	*in vitro*, human	U87, U251	Gulinaer et al.^121^
miR-141	down	TGF-β2	*in vitro*, human	U251, U87, U118, LN18	Peng et al.^122^
miR-200b	down	ZEB2	*in vitro*, human	U251, U87	Li et al.^123^
miR-138	down	IGF2BP2	*in vitro*, human	Res186, Res259	Yang et al.^124^
miR-22	down	SNAIL1	*in vitro*	U-87, U-118 MG, M059K, Hs 683	Zhou et al.^125^
miR-508-5p	down		*in vitro*, human		Liu et al.^126^
miR-132	down	TTK	*in vitro*, human	U-87	Thunshelle et al.^127^
miR-101-3p	down	TRIM44	*in vitro*	U87MG, U251MG, U118MG, T98	Li et al.^128^
miR-378	down	IRG1	*in vitro*, human	SHG44, A172, LN229, LN18	Shi et al.^129^
miR-758-5p	down	ZBTB20	*in vitro*, human	U118, LN-299, H4, A172, U87-MG, U251	Liu et al.^130^
miR-27b	up	Spry2	*in vitro*, human	U87, U251, SHG44	Liu et al.^131^
miR-424	down	KIF23	*in vitro*, human	A172, SHG-44, T98, LN18, LN229	Zhao et al.^132^
miR-154-5p	down	PIWIL1	*in vitro*, human	U251, U87, A172, LN229, SNB19, LN308	Wang et al.^133^
miRNA-132	down	MMP16	*in vitro*, human	A172, SHG44, U87	Wang et al.^134^
miR-200b	down	CREB1	*in vitro*, human	U87, SF126, U251, SF767	Peng et al.^135^
miR-374b	down	EGFR	*in vitro*, human	U251, U87	Pan et al.^136^
miR-622	down	ATF2	*in vitro*, human	U87, U251, A172, U118, LN229	Zhang et al.^137^
miR-29a	up	PTEN	*in vitro*, human	U87, U251, LN229	Zhao et al.^138^
miR-422a	down	IGF1, IGF1R	*in vitro*, human	U87, U251	Wang et al.^139^
miR-139-3p	down	NOB1	*in vitro*, human	U251, U87MG, TJ905, SHG44	Shi et al.^140^
miR-491	down	Wnt/β-catenin	*in vitro*, human	s LN18, LN229	Meng et al.^141^
miR-1290	up	LHX6	*in vitro*, *in vivo*, human	LN-229, U87	Yan et al.^142^
miR-7	down	EGFR	*in vitro*, *in vivo*, human	U-87MG, U-118MG	Wang et al.^143^
miR-351	down	NAIF1	*in vitro*	U87, U251	Wu et al.^144^
miR-150-3p	down	SP1	*in vitro*, human	U251, U87MG, A172, SWO-38, SHG44	Tan et al.^145^
miR-133b	down	Sirt1	*in vitro*, human	U87	Li et al.^146^
miR-30b-3p	up	RECK	*in vitro*, human	SHG44, U251, U87 A172	Jian et al.^147^
miR-144	down	FGF7, CAV2	*in vitro*, human	U251, LN229, LN18	Liu et al.^148^
miR-30a	down	Wnt5a	*in vitro*, human	T98G, SHG44, U251, U87, U373	Zhang et al.^149^
miR-221/222	up	TIMP2	*in vitro*, human	U87, U251, SHG-44, BT325, A172	Yang et al.^150^
hsa-mir-127	up	REPIN1	*in vitro*	U87, LN-229	Wang and Lin^151^
miR-202	down	MTDH	*in vitro*, human	A172, U87, U251, U373, LN229	Yang et al.^152^
miR-219	down	SALL4	*in vitro*, human	A172, U87, U251, U373	Jiang et al.^153^
miR-204-5p	down	RAB22A	*in vitro*, human	LN-229, U87	Xia et al.^154^
miR-188	down	IGF2BP2	*in vitro*, human	U87, U251, U118, LN229, LN18	Ding et al.^155^
miR-200c	down	MSN	*in vitro*, *in vivo*, human	H4, U251	Qin et al.^156^
miR-320	down	E2F1	*in vitro*, human	U251, SHG-44	Sun et al.^157^
miR-139-3p	down	MDA9/syntenin	*in vitro*, human	U87MG, U251MG, U118, A172	Tian et al.^158^
miR-637	down	Akt1	*in vitro*, human	U251, U87	Que et al.^159^
miR-376a	down	SP1	*in vitro*, human	U138, U251, LN229, T98	Li et al.^160^
miR-590-3p	down	ZEB1, ZEB2	*in vitro*, human	U87MG, A172	Pang et al.^161^
miR-16	down	SALL4	*in vitro*, human	U251, U87	Han et al.^162^
miR-98	down	RKIP	*in vitro*, human	U251, U87, SHG44	Chen et al.^163^
miR-370	down	β-catenin	*In vitro*, human	U251, U87	Lu et al.^164^
miR-139-5p	down	ZEB1, ZEB2	*in vitro*, human	U87, A172	Yue et al.^165^
miR-217	up	YWHAG	*in vitro*, *in vivo*, human	U87 MG, U118 MG, U251, U87	Wang et al.^166^
miR-548b	down	MTA2	*in vitro*, *in vivo*, human	U87, T98G, U373, LN229, SNB19, U251	Pan et al.^167^
miR-663	down	TGF-β1	*in vitro*, human	A172, U87	Zhang et al.^168^
miR-489	down	SPIN1	*in vitro*, *in vivo*, human	U87, T98, U251	Li et al.^169^
miR-10b	up	TGF-β1	*in vitro*, *in vivo*, human	U87, U251	Ma et al.^170^
miR-20a	up	TIMP-2	*in vitro*, *in vivo*, human	U87	Wang et al.^171^
miR-106a	up	TIMP-2	*in vitro*, *in vivo*, human	U87	Wang et al.^171^
miR-146b	up	MMP16	*in vitro*	U87, U373, U138, U118, SW1783, SW1088	Xia et al.^172^
miR-203	down	GAS41/miR10b	*in vitro*	U87, HNGC2	Pal et al.^173^
miR-204	down	ezrin	*in vitro*, human	U87, U118, U138, U87, SW1088, SW1783, CCF-STTG1	Mao et al.^174^
miR-873	down	IGF2BP1	*in vitro*, human	A172, T98G, U87, U373, U251, U138	Wang et al.^175^
miR-144-3p	down	FZD7	*in vitro*, human		Cheng et al.^176^
miR-124	down	PPP1R3L	*in vitro*, human	U251, U373	Zhao et al.^177^
miR-351	down	NAIF1	*in vitro*	U87, U251	Wu et al.^178^
hsa-miR-9	down	MAPKAP	*in vitro*, human	T98G, U251, SF295	Ben-Hamo et al.^179^

miR-382 inhibits the development and metastasis of GBM and could be a novel therapeutic target to increase efficacy of GBM therapy.^180^ Tripartite pattern containing 44 (TRIM44) is a member of the TRIM family of proteins and is involved in various disorders, including viral infections, developmental abnormalities, and neurodegenerative diseases.^181^, ^182^, ^183^ The presence of an E3 site in their structure allows E3 ubiquitin ligase activity, which can be regulated after translation. Earlier studies found that TRIM44 over-expression was present in many cancers, which stimulated development, proliferation, and progression of the cell cycle. Furthermore, over-expression of *TRIM44* could promote invasion and migration of cancer cells and increase the metastatic potential of the tumor.^184^, ^185^, ^186^ In conclusion, inhibiting the expression of TRIM44 could be useful in the management of tumor metastasis and also inhibit tumor growth.

In one study, RT-PCR was used to measure miR-623 expression levels in GBM tissue. miR-623 up-regulation and its effects on proliferation, invasion, and migration of malignant cells was evaluated using transwell, colony formation, and MTS assays.^112^ Moreover, a subcutaneous mouse xenograft model was used to evaluate the effects *in vivo*. A dual-luciferase reporter assay was used to confirm miR-623-TRIM44 binding and western blotting to assess the effect of miR-623 on markers of the EMT. miR-623 was down-regulated in cell lines and samples from GBM patients. Over-expression of miR-623 or suppression of TRIM44 inhibited GBM cell proliferation, invasion, and migration. On the other hand, inhibition of miR-623 increased *TRIM44* expression, the EMT, and the consequent progression of GBM. Expression of TRIM44 was suppressed by direct binding of miR-623 to the 3′ UTR region. Furthermore, in nude mice with a GBM xenograft, systemic administration of a miR-623 mimic inhibited tumor development and suppressed expression of TRIM44 protein. They also verified that high expression of miR-623 or low expression of *TRIM44* inhibited the proliferation and migration of the glioma cell lines U251MG and LN229. They concluded that miR-623 could decrease EMT triggered by *TRIM44* by direct targeting of the TRIM44 3′ UTR and could be a new therapeutic target for GBM treatment.^112^

Studies have found that miR-140 is often dysregulated in several cancers. Down-regulation of miR-140 has been identified in adenocarcinoma of the pancreatic duct,^187^ lung cancer,^188^^,^^189^ colorectal cancer,^190^ ovarian cancer,^191^ esophageal cancer,^192^ and cancer of the tongue.^193^ On the other hand, miR-140 has also been found to be highly expressed in some other tumors, such as spinal chordoma^194^ and breast cancer.^195^ Down-regulation of miR-140 enhanced invasion and EMT in esophageal cancer cells.^192^ Besides, up-regulation of miR-140 was associated with growth inhibition in tumor cells and less metastasis in hepatocellular carcinoma.^196^ miR-140 was found to act as a tumor suppressor in previous studies, indicating that restoration of the expression of miR-140 may help in cancer therapy. A variety of miR-140 target genes have been identified, such as iASPP,^187^ ATP6AP2,^188^ ATP8A1,^189^ VEGFA,^190^ PDGFRA,^191^ Slug,^192^ and IGF-1R.^197^

Nevertheless, miR-140 targets have not often been reported in glioma patients. One study reported that miR-140 acted in glioma cells by adversely modulating a newly discovered target, called ADAM9. A disintegrin and metalloproteinases (ADAMs) belong to the metzincin superfamily of matrix metalloproteinases.^198^ ADAM9 belongs to the ADAM family and contains an N-terminal pro-domain, which is accompanied by a metalloprotease domain, a disintegrin domain, a cysteine-rich region, a transmembrane domain, an epidermal growth factor similar region, and a tail with a possible SH3 ligand domain located in the cytoplasm.^199^^,^^200^ Accumulating evidence has shown the over-expression of ADAM9 in several human cancers, including renal cell carcinoma,^201^ prostate cancer,^202^ breast cancer,^203^ hepatocellular carcinoma,^204^ and pancreatic cancer.^205^ ADAM9 was found to be highly expressed in glioma samples and promoted migration and invasion in glioma cells.^206^^,^^207^ As a result, ADAM9 could be a potential therapeutic target for treatment of human tumors.

In a study by Liu et al., the miR-140 expression level was evaluated in glioma patients and the effects of miR-140 on proliferation, migration, and invasion of tumor cells in glioma tissue.^120^ They found significant down-regulation of miR-140 in glioma patient samples, which was associated with poor Karnofsky performance score (KPS) and World Health Organization (WHO) grades. Restoration of miR-140 expression significantly reduced the ability of glioma cells to proliferate, invade, and migrate. The ADAM9 gene was identified to be a new direct target gene of miR-140 in glioma patients. Moreover, silencing of ADAM9 mimicked the activity of miR-140 as a tumor-suppressor gene in glioma. Meanwhile, over-expression of ADAM9 abrogated the inhibitory effect of miR-140 in glioma cells. They concluded that miR-140 deregulation played a major role in the development of glioma and served as a tumor suppressor in glioma pathogenesis. miR-140 could inhibit expression of ADAM9 and thereby suppress proliferation, invasion, and migration of glioma cells. As a result, miR-140 could be a new potential target to develop promising treatment approaches for glioma patients.^120^

Deregulation of miR-133b has been found to play important roles in many human cancers.^208^, ^209^, ^210^ This process was mediated by direct targeting of the receptor tyrosine kinase MET.^211^ Previous studies have reported that miR-133b may play a key role in glioma development.^212^ In a study by Wang et al., miR-133b was shown to be significantly down-regulated *in vitro* in GBM cell lines and could directly target the human-Ether-à-Go-Go-related gene (hERG) channel, which increased apoptosis in U251 glioma cells after treatment with arsenic.^212^

In a study by Li et al., the ability of miR-133b to regulate glioma cell proliferation and invasion was investigated.^146^ miR-133b was considerably under-expressed in glioma samples in comparison with adjacent healthy tissue. Sirt1 was verified as a new direct target of miR-133b in U87 glioma cells. Up-regulation of miR-133b was associated with lower expression of Sirt1 and suppressed the invasion and proliferation of U87 glioma cells. This phenomenon could be partially rescued by induced over-expression of Sirt1. Furthermore, Sirt1 mRNA was found to be considerably up-regulated in glioma samples, compared with adjacent healthy tissue, and had an inverse correlation with the level of miR-133b in tumor cells. In conclusion, the role of miR-133b in modulating the development and metastasis of gliomas could be mediated by Sirt1 expression and could be a therapeutic target for glioma patients.^146^

Silent information regulator 1 (Sirt1) is a member of the family of the mammalian sirtuin proteins. Sirt1 serves as a histone deacetylase enzyme that is dependent on nicotinamide adenine dinucleotide (NAD^+^) and plays a key role in regulating the cell cycle and gene transcription.^213^ Sirt1 has been implicated in many cell biology processes, such as cell cycle progression, metabolism, proliferation, cell death pathways, differentiation, and senescence.^214^, ^215^, ^216^, ^217^, ^218^, ^219^, ^220^ Sirt1 has been found to have dual effects in human cancers. In one study by Kim et al., Sirt1 was found to serve as a tumor suppressor in breast cancer patients, suggesting that down-regulation of Sirt1 would reflect an unfavorable prognosis and lead to more metastasis.^221^ On the other hand, Sirt1 was over-expressed in colon cancer and was associated with mutations of P53 and the tumor, node, and metastasis (TNM) stage.^222^ According to Lu et al., Sirt1 reduced the development of gastric cancer by inhibiting the activation of NF-κB and STAT3,^223^ and they concluded that Sirt1 plays a complex role in cancer development. Whether Sirt1 could function as an oncogene in the pathogenesis of glioma was a major question in recent studies. Sirt1 increased proliferation and suppressed apoptosis in glioma tissue.^224^ In addition, Sirt1 silencing was found to promote the sensitivity of the CD133^+^ cells in glioma tissue to radiotherapy, both *in vivo* and *in vitro*.^225^ SIRT1 also facilitated p53 activity in the tumorigenesis of CNS stem cells.^226^

miR-27b is a ncRNA with a regulatory role in the development of many tumors. Wan et al. reported that miR-27b expression was significantly down-regulated in non-small cell lung cancer (NSCLC) cell lines, and up-regulation of miR-27b expression was associated with significant inhibition of the invasion and proliferation of tumor cells.^227^ This suggests that miR-27b acts as a tumor suppressor in the pathogenesis of NSCLC. Several studies have reported that miR-27b inhibited the progression and development of different cancers, such as neuroblastoma, prostate, and colorectal cancer.^228^, ^229^, ^230^ However, some other studies have suggested that miR-27b could enhance tumorigenesis. In one study by Jin et al., miR-27b was significantly over-expressed in other malignancies, such as breast cancer. Silencing of miR-27b expression significantly suppressed the development of breast cancer.^231^

miR-27b was significantly up-regulated in glioma samples in comparison with adjacent healthy brain tissue.^131^ Moreover, miR-27b has been reported to be highly expressed in samples from glioma patients and in glioma cell lines (U251, SHG44, and U87) compared with healthy astrocytes and adjacent brain tissue. Spry2 was verified as a new miR-27b target in glioma cell lines (U251), and the level of expression of Spry2 protein was inversely correlated with miR27b in glioma cells. In addition, miR-27b suppression and Spry2 up-regulation both inhibited glioma cell invasion. On the other hand, low expression of Spry2 abolished the miR-27b inhibitory effect on glioma cell invasion. The result of this study showed that miR-27b could directly inhibit the expression of Spry2 and increase glioma invasion. The results also suggest that miR-27b could be a molecular target to inhibit glioma metastasis and invasion.^131^

Sprouty homolog 2 (Spry2) belongs to the Sprouty family (named for *Drosophila* development) and contains a carboxy-terminal region that is cysteine-rich, which plays a crucial role in the suppression of receptor tyrosine kinase signaling.^232^ Spry2 contributes to the modulation of tumor cell invasion by regulating the mitogen-activated protein kinase (MAPK) signaling axis.^233^^,^^234^ The Spry2 protein level was recently shown to be notably down-regulated in glioma patients with a more invasive tumor type, confirming the regulatory function of Spry2 in glioma invasion.^235^

Table 1 lists various metastasis-related miRNAs reported to be involved in GBM.

## Long non-coding RNAs and metastasis in glioma

The lncRNA called FOXD2-AS1 (NR_026878) is located on chromosome 1p33 and contains 2,527 nt. It was discovered to be highly expressed in gastric cancer.^236^ In other studies, it was shown that FOXD2-AS1 could be a molecular marker for some cancers. FOXD2-AS1 expression was correlated with invasion, migration, and low apoptosis of cancer cells and a poor outcome of patients with these tumors.^237^, ^238^, ^239^, ^240^ FOXD2-AS1 could lead to glioma progression by modulating the PI3K/AKT signaling pathway and also the miR-185-5p/high-mobility group 2 (HMGA2) axis.^241^

One study was carried out to measure the expression of CDK2, P21, cyclinE1, matrix metalloproteinase 7 (MMP7), MMP9, neural and epithelial cadherins, vimentin, miR-506-5p, and FOXD2-AS1.^242^ mir-506-5p was found to be a direct target of FOXD2-AS1 by a luciferase reporter assay. They found that FOXD2-AS1 expression was significantly higher in glioma cells, particularly in the U251 cell line. Moreover, low expression of FOXD2-AS1 resulted in significant reduction of tumor invasion, cell migration and proliferation, and suppression of the EMT. In addition, FOXD2-AS1 could regulate the expression level of P21, cyclinE1, MMP7 and MMP9, and CDK2. The possible underlying mechanism was suggested to be that FOXD2-AS1 down-regulated the expression of miR-506-5p, an anti-oncogene in several human cancer types. Over-expression of mir-506-5p and transfection of FOXD2-AS1 had the opposite effects, in that high expression of miR-506-5p inhibited proliferation, invasion, and migration as well as EMT. In conclusion, FOXD2-AS1 could facilitate the EMT and the subsequent metastasis of glioma cells through inhibiting miR-506-5p. As a result, FOXD2-AS1 could be a new target in glioma treatment.^242^

The lncRNA cancer susceptibility candidate 2 (CASC2) located on chromosome 10q26 has been found to act as an anti-oncogene.^243^ Over-expression of CASC2 could target mir-193a and mir-21 and inhibit malignant behavior in glioma.^244^^,^^245^ The down-regulation of CASC2 was correlated with a shorter survival time in glioma patients.^246^ Although researchers are investigating the function and clinical implications of CASC2, its molecular mechanism remains understudied and needs more research. miR-18a-5p has been identified as an oncogene in several tumor types^247^ and is likely to play the same role in glioma.^248^^,^^249^

In a study by Wang et al., they showed that CASC2 could act as a tumor suppressor.^250^ CASC2 over-expression led to more apoptosis and increased expression of E-cadherin (but not N-cadherin) and vimentin in A172 and T98 glioma cell lines. Furthermore, over-expression of CASC2 suppressed cell migration, viability, and colony-forming ability in T98 and A172 cells. It was concluded that miR-18a was a downstream target of CASC2, and CASC2 expression was inversely correlated with mir-18a levels.^250^

Another highly conserved lncRNA in mammals is metastasis-associated lung adenocarcinoma transcript-1 (MALAT1), which is composed of about 8,000 nt.^251^ Studies reported that MALAT1 was highly expressed in different types of tumors, such as gastric adenocarcinoma, squamous cell carcinoma, and hepatocellular carcinoma, and played a crucial role in the development of these cancers.^252^, ^253^, ^254^ Furthermore, MALAT1 was associated with hyperproliferation and metastasis in lung cancer via regulating factors, such as p53 and c-MYC, and also played a role in the EMT.^255^ Furthermore, MALAT1 was associated with progression of glioma tumors,^256^ but the exact mechanism of MALAT1 in glioma is still uncertain. Ras-related protein1 (Rap1) is a globally expressed small guanosine triphosphatase (GTPase) that transduces signals from different receptors. Cellular functions, such as adhesion, polarity, and migration,^257^ can be modulated by the two RAP1 isoforms, Rap1A and Rap1B. These molecules have been reported to affect invasion, metastasis, and proliferation in different cancer types, including ovarian and colorectal cancer.^258^, ^259^, ^260^

Li et al. evaluated the role of MALAT1 and its mechanisms in glioma cells.^261^ In this study, they measured the expression levels of miR-101**,** Rap1B mRNA, and MALAT1 in U87 and U251 glioma cells. They found that both Rap1B and MALAT1 were up-regulated, while miR-101 expression was down-regulated in U87 and U251 cells. Knockdown of either Rap1B or MALAT1 reduced cell proliferation and promoted apoptosis. There was a correlation between the expression of Rap1B and MALAT1, and it was suggested that MALAT1 increased the expression of Rap1B through sponging miR-101 in U87 and U251 cells. Silencing of MALAT1 in glioma cell lines reduced proliferation and enhanced apoptosis, while suppression of miR-101 or over-expression of Rap1B had the opposite effects on proliferation and apoptosis. This finding showed a novel regulatory axis consisting of MALAT1, Rap1B, and miR-101, which could serve as a target in glioma treatment.^261^

mir-124 has also been found to be a potential target of MALAT1. miR-124 is widely distributed in brain tissue and also plays a major role in a number of human cancers.^262^^,^^263^ There is increasing evidence that miR-124 could inhibit invasion and suppress tumor growth in different cancer types, such as colorectal cancer, breast cancer, renal cancer, and cervical cancer.^264^, ^265^, ^266^, ^267^ Feng et al. suggested that the expression of miR-124 in breast cancer was lowered by the binding of MALAT to miR-124 and thus MALAT1 could be a potential endogenous regulator.^268^ miR-124 was identified as a direct target of MALAT1. It was revealed that the higher expression of MALAT1 in glioma tumors correlated with lower miR-124 expression. Previous research showed that ZEB2 was associated with pathologic and clinical features of human tumors, such as tumor grade, patient overall survival, patient prognosis, and neoplastic progression.^269^, ^270^, ^271^ In a study by Qi et al., it was found that the migration, invasion, and proliferation of glioma cells may be suppressed by down-regulation of ZEB2. In addition, down-regulation of ZEB2 resulted in G1/S cell-cycle arrest and more apoptosis in glioma cells.^272^ In different human tumors, ZEB2 was found to regulate various miRNAs, such as miR-132, miR-101, miR-144, and miR-141.^273^, ^274^, ^275^, ^276^ In a recent study, MALAT1 was found to affect ZEB2 expression via sponging miR-200 in clear-cell renal carcinoma.^277^

In a study by Cheng et al., the expression of MALAT1 was found to be increased in human glioma cells and tissues, and it was proposed to act as a functional oncogene. Moreover, MALAT1 was associated with poor outcomes in glioma patients, and silencing of MALAT1 resulted in reduced cell proliferation and caused cycle arrest and apoptosis. In addition, MALAT1 expression was correlated with glioma tumor volume. MALAT1 silencing reduced the tumor volume, while miR-24 had the opposite effect on tumor volume. miR-124 could reverse the silencing of MALAT1 and the subsequent tumor-suppressor effect in different human cancer xenografts. It has been shown that ZEB2 serves as a direct target of miR-124 and ZEB2 expression was down-regulated by miR-124 and also by MALAT1 over-expression-induced ZEB2 expression. In conclusion, this study revealed a new MALAT1/mir-124/ZEB2 axis that was correlated with glioma progression and could be a new therapeutic target in glioma.^278^

Long intergenic non-protein coding RNA689 (*LINC00689*) has been found to be involved in various human cancers, A clinical study carried out in Northern Han Chinese individuals showed that *LINC00689* was a gene related to obesity.^279^ The stress and tumor necrosis factor alpha (TNF-α)-activated open reading frame (ORF) micropeptide (STORM) peptide is encoded by *LINC00689* and can act in a similar manner to signal recognition particle 19 (SRP19) in controlling gene expression in eukaryotic cells.^280^ *miR-338-3p* was found to be a tumor suppressor in several cancer types.^281^, ^282^, ^283^ miR-338-3p inhibited migration and induced apoptosis in gastric cancer cells via affecting its target, protein-tyrosine phosphatase 1B (PTP1B).^281^ Furthermore, miR-338-3p suppressed the progression of hepatocellular carcinoma (HCC) cells and inhibited the Warburg effect via restoration of the activity of pyruvate kinase L/R (PKLR).^282^ mir-338-3p acts as an anti-oncogene by suppressing GBM invasion and proliferation and reducing ATP synthesis by targeting the pyruvate kinase M2 (PKM2)-β-catenin axis.^283^

The lncRNA *SBF2-AS1* was found to sponge *miR-338-3p* in GBM cells.^284^ Afterward, PKM2 was reported to be a downstream target of the *LINC00689-miR-338-3p* axis in glioma patients. PKM2 was found to enhance proliferation, glycolysis, and metastasis of HCC tumor cells. In addition, PKM2 expression was correlated with glucose metabolism and malignant properties in glioma cells.^285^^,^^286^

In a study by Liu et al., the expression of LINC00689 was found to be higher in glioma tissue in comparison with normal tissue, on the basis of the GSE dataset (GEO: GSE4290). Then, they demonstrated experimentally that LINC00689 was highly expressed in glioma tissue and cell lines and LINC00689 expression was correlated with a larger tumor size (particularly >3 cm), poor prognosis, and a low KPS score.^287^ LINC00689 knockdown led to suppression of glioma cell proliferation, migration, and glycolysis. Moreover, LINC00689 knockdown was associated with significant inhibition of glioma tumor growth *in vivo*. LINC00689 enhanced the expression of PKM2 through a direct interaction with miR-338-3p, suggesting that LINC00689 plays a competing endogenous RNA (ceRNA) role in glioma. The effects of LINC00689 knockdown on proliferation, invasion, migration, and glycolysis of glioma cells could be abrogated by PKM2 restoration. In conclusion, the LINC00689/miR-338-3p/PKM2 axis plays a role in glioma progression.^287^ Table 2 lists some metastasis-related lncRNAs reported to be involved in GBM.

**Table 2:** Metastasis-related lncRNAs in GBM

lncRNAs	Expression status	Targets	Model (*in vitro*, in vivo, human)	Type of cell line	Ref
FOXD2-AS1	up	miR-506-5p	*in vitro*	U251, SHG44, LN229, T98G	Zhao et al.^242^
NBAT1	down	miR-21/SOX7	*in vitro*, human	AM38, Gli-6, GSC11, A172	Guan et al.^288^
HCG11	down	miR-4425/MTA3	*in vitro*, *in vivo*, human	A172, U251, U87MG, U118	Zhang et al.^289^
MEG3	down	miR-96-5p/MTSS1	*in vitro*, human	GSC11, M059J, D54	Zhang and Guo^290^
DANCR	up	miR-33a-5p	*in vitro*, *in vivo*, human	U87, U251, T98G, LN22 9	Yang et al.^291^
lncRNA001089	down		*in vitro*, *in vivo*, human	U251	Perez-Laguna et al.^292^
HOTTIP	up	miR-101/ZEB1	*in vitro*, human	U87, U251	Zhang et al.^293^
CASC19		miR-454-3p/RAB5A	*in vitro*, human		Wu et al.^294^
MALAT1	up	miR-124/ZEB2	*in vitro*, *in vivo*, human	U251	Cheng et al.^295^
GHET1	up	miR-216a	*in vitro*	U251	Cao et al.^296^
CASC2	down	miR-18a	*in vitro*, *in vivo*, human	T98, A172	Wang et al.^250^
MALAT1	up	Rap1B, miR-101	*in vitro*, human	U87, U251	Xiang et al.^297^
H19	up	miR-29a	*in vitro*, *in vivo*, human	U87MG	Jia et al.^298^
MALAT1	up	miR-101	*in vitro*	U251, U87	Li et al.^261^
XIST	up	miR-429	*in vitro*, *in vivo*, human	A172, U251	Cheng et al.^299^
TSLNC8	down		*in vitro*, human	BE-2C, BT325, SHG-44, CHG-5 U25-MG, SWO38	Chen and Yu^300^
lncRNA-LYPLAL1-2	down	miR-127/YWHAG	*in vitro*, *in vivo*, human	U87, U251	Zheng et al.^301^
LINC00961	down		*in vitro*, human	U251, A172, U-118, U87	Abedi-Gaballu et al.^302^
UBE2R2-AS1	down	miR-877-3p/TLR4	*in vitro*, human	U251, A-172, U373, U87-MG	Xu et al.^303^
FOXD2-AS1	up	miR-185	*in vitro*, human	U251,LN18, T98G, A172, LN22	Dong et al.^304^
SNHG18		ENO1	*in vitro*	M059J, M059K, U87	Zheng et al.^305^
TP73-AS1	up	miR-124	*in vitro*, human	U87, U118, U251, U373, SHG-44	Xiao et al.^306^
SPRY4-IT1	up		*in vitro*, human	U251, SF295	Liu et al.^307^
GAS5	down	miR-18a-5p	*in vitro*, *in vivo*, human	U251, U87	Liu et al.^308^
ZEB1-AS1	up	miR-200c/141-ZEB1	*in vitro*, *in vivo*, human	U87, U251, LN18, U118, T98G	Meng et al.^309^
LINC01426	up	PI3K/Akt	*in vitro*, human	PG1, A172, LN229, U251, LN118, H4	Wang et al.^310^
LINC00689	up	miR-338-3p/PKM2	*in vitro*, *in vivo*, human	U87, U251	Liu et al.^287^
NEAT1	up	miR-139-5p/CDK6	*in vitro*, *in vivo*, human	U251, SHG-44, TJ905	Wu et al.^311^
MALAT1	up	miR-199a/ZHX1	*in vitro*, *in vivo*, human	U87-MG, U251, T98G, A172	Liao et al.^312^
LIFR-AS1	down	miR-4262/NF-κB	*in vitro*, human	A172, U87, U251, LN229	Ding et al.^313^
MALAT1	up		*in vitro*, human	primary	Ma et al.^256^
MALAT1		NF-κB, p53	*in vitro*, *in vivo*	U87, A172, U251	Voce et al.^314^
XIST	up	miR-133a/SOX4	*in vitro*	U251	Luo et al.^315^
HOXA11-AS	up	miR-130a-5p-HMGB2	*in vitro*, *in vivo*, human	U251, U87MG	Xu et al.^316^
MALAT1	down	ERK/MAPK	*in vitro*, *in vivo*, human	U87, U251	
MALAT1	down	miR-155	*in vitro*, human	U87, SHG139	Cao et al.^317^
EGOT	down		*in vitro*, human	A172, U251, U87, SHG44,	Wu et al.^318^
HOXD-AS1	up	miR-130a	*in vitro*, human	U87, U251	Chen et al.^319^
GAS5-AS1	down	miR-106b-5p/TUSC2	*in vitro*, *in vivo*, human	U251	Huang et al.^320^
PVT1	up	UpF1	*in vitro*, human	U87, LN229	Lv et al.^321^
NEF	down	TGF-β1	*in vitro*, human	Hs 683, CCD-25Lu	Wang et al.^322^
SAMD12-AS1	up	P53	*in vitro*, human	U251, U87, T98-G, A172	Jia et al.^323^
FOXD2-AS1	up	miR-185-5p/HMGA2	*in vitro*, *in vivo*, human	U87, A172, U251, T98G	Ni et al.^241^
MEG3	down	miR-21-3p	*in vitro*	U87, U251	Qin et al.^324^
ANCR	up	EZH2, PTEN	*in vitro*, human	U87, U251, SHG44, U118	Cheng et al.^325^
Linc01116	up	miR-31	*in vitro*, *in vivo*, human	SHG-44, U87, U25, U118 MG	Zhang et al.^326^
LSINCT5	up	miR-451	*in vitro*, human	GL15	Liu et al.^327^
lincRNA-p21		miR-34c	*in vitro*, *in vivo*	U87, U251	Yang et al.^328^
AWPPH	up	HIF1α	*in vitro*, human	Hs 683, CCD-25Lu	Zhang et al.^329^
MACC1-AS1	up	MACC1	*in vitro*, human	U251, T98G, A172, SHG44	Zheng et al.^330^
H19	up	miR-342/Wnt5a/β-catenin	*in vitro*, *in vivo*, human	A172, LN229, U251	Zhou et al.^331^
AC016405.3	down	miR-19a-5p, TET2	*in vitro*, human	U87MG	Ren and Xu^332^
MIR4697HG	down	miR-766-5p/PRR12	*in vitro*	U343, Hs683, LN25, A172, LN18, U87, U251	Mao et al.^333^
LINC00466	up	miR-508/CHEK1	*in vitro*, human	A172, T98G, LN299, U251, LN18	Li et al.^334^
SNHG16	up	miR-490/PCBP2	*in vitro*, human	T98G, U251	Kong et al.^335^
MALAT1	up		*in vitro*, human	primary	Argadal et al.^336^
LINC01614	up	miR-383/ADAM12	*in vitro*, human	LN18, U251, T98G, LN229, A172	Wang et al.^250^
MALAT1	up	miR-384/GOLM1	*in vitro*, *in vivo*, human	SHG-44, LN229	Alcon-Giner et al.^337^
GAPLINC	up	miR-331-3p	*in vitro*, human	T98G, U251, LN18, LN229, A172	Chen et al.^338^
H19	up	miR-140, iASPP	*in vitro*, human	U373, A172, U251, T98G, U87MG	Zhao et al.^339^
FTH1P3	up	miR-224-5p/TPD52	*in vitro*, human	U251	Zhang et al.^340^
HOXA11-AS	up	miR-214-3p/EZH2	*in vitro*, *in vivo*, human	U251, U87, LN229, SHG-44, A172	Xu et al.^341^
SNHG7	up	miR-5095, Wnt/β-catenin	*in vitro*, *in vivo*, human	A172, U87, T98G, SHG44	Ren et al.^342^
UCA1	up	miR-182, iASPP	*in vitro*, human	U373MG, T98MG, SWO38, U251, SHG44	He et al.^343^
LINC01260	down	CARD11, NF-κB	*in vitro*, *in vivo*, human	U251	Wu et al.^344^
RP5-833A20.1	down	microRNA-382-5p, NFIA	*in vitro*, human	U251	Kang et al.^345^
UCA1		miR-204-5p/ZEB1	*in vitro*, *in vivo*	SHG44, U87MG	Liang et al.^346^
SNHG5	up	miR-205-5p/ZEB2	*in vitro*, human		Meng et al.^347^
MALAT1	up		*in vitro*	SHG139, SHG139S	Han et al.^348^
LINC-PINT	down	Wnt/β-catenin	*in vitro*, *in vivo*	U87, LN229, U373, A172, U251, T98, U118	Zhu et al.^349^

## circRNAs and metastasis in glioma

Previous studies have confirmed the regulatory role of circRNAs in tumor malignancy and metastasis to adjacent tissues.^350^ An increasing number of studies suggest that circRNAs can cause tumor drug resistance and increase metastasis and recurrence.^351^ Certain specific circRNAs, such as circ_0067934,^352^ circ_0014359,^353^ and circZNF264,^354^ have been found to be involved in glioma progression. One theory is that circRNAs may act as ceRNAs to sponge miRNAs and thereby reduce the aggressive malignant properties of cancer cells by regulation of mRNA expression.^355^ For example, circFOXO3 encouraged invasion and cell proliferation by sponging both miR-182 and miR-433 in glioma.^356^ Xing et al. reported that circFOXO3 could promote esophageal squamous cell carcinoma (SCC) progression via the miR-23a-3p/PTEN axis.^357^ Li et al. found that increased expression of circU2AF1 promoted glioma progression and was correlated with a poor clinical prognosis.^358^ A study by Lei et al. reported that circ_0076248 could encourage glioma progression by increasing SIRT1 due to the sponging of miR-181a.^359^ Another study suggested that circFBXW7 could be a therapeutic target to inhibit glioma progression.^360^ Yang and colleagues used RNA sequencing to determine circRNA profiles in glioma samples compared with normal adjacent tissue and found that circFBXW7 was expressed higher in normal samples compared with glioma. Higher levels of circFBXW7 were correlated with longer patient survival and a better prognosis.^79^

Gao et al. evaluated the function of circFBXW7 in glioma and its underlying mechanism.^361^ The expression of circFBXW7, miR-23a-3p, and PTEN was measured by qRT-PCR in glioma tissue and cell lines. The proliferation of glioma cells was measured by Cell Counting Kit 8 (CCK8) assay. The migration and invasion of glioma cells were measured by transwell assays. Possible interactions between circFBXW7, PTEN, and miR-23a-3p were evaluated using a dual-luciferase reporter assay. Western blotting measured the expression of these proteins. A mouse xenograft model of glioma was used to investigate circFBXW7 function *in vivo*. They found that circFBXW7 expression was significantly lower in glioma cell lines and tumor tissue compared with normal tissue. High expression of circFBXW7 had an inhibitory effect on glioma cell migration, proliferation, and invasion. Furthermore, miR-23a-3p was shown to be a direct target of circFBXW7 using bioinformatics analysis and dual-luciferase reporter assay. The binding of miR-23a-3p to the PTEN 3′ UTR might be inhibited by the activity of circFBXW7. They concluded that circFBXW7 reduced glioma metastasis and proliferation by directly sponging miR-23a-3p, thus increasing PTEN. circFBXW7 up-regulation inhibited glioma growth and metastasis *in vivo*. Over-expression of circFBXW7 resulted in decreased expression of Ki67, miR-23a-3p, and N-cadherin but increased levels of PTEN and E-cadherin, which was in agreement with the *in vitro* findings. They suggested that circFBXW7 could be a new therapeutic and diagnostic target in glioma patients.^361^

New studies have reported that the inhalational anesthetic sevoflurane (Sev) could suppress the progression of tumors through modulating miRNAs. Sun et al. found that Sev inhibited the invasion and migration of colorectal cancer cells by modulating the miRNA-34a/ADAM10 axis.^362^ A study by Gao et al. reported that Sev could inhibit metastasis and proliferation of glioma cells by targeting the miRNA-124-3p/ROCK1 axis.^363^ Recently, Xie et al. reported that miR-628-5p could suppress cell proliferation in glioma.^364^ However, the exact mechanism of how Sev affects miR-628-5p and the progression of glioma are not yet clear.

Magnesium transporter 1 (MAGT1) has been shown to be associated with several human cancers. Zheng et al. reported that high expression of MAGT1 was a poor prognostic indicator in colorectal cancer.^365^ Wang et al. showed that miRNA-199a-5p suppressed progression of glioma by inhibiting MAGT1.^366^

Li et al. evaluated the effect of Sev on glioma progression using bioinformatics analysis and experimental studies.^367^ They used flow cytometry for apoptosis; western blotting for protein levels of hexokinase 2 (HK2), MAGT1, Bcl-2, and BCL2-associated X (Bax) in glioma samples; and CCK8 assay for cell viability. The transwell assay measured cell migration and invasion. Colorimetric assay kits were utilized to measure lactate synthesis and glucose metabolism. The levels of circRNA1656 (also known as circ-0002755) and mir628-5p were measured by qRT-PCR, and the interactions between miR-628-5p, circ-0002755, and MAGT1 were confirmed by dual-luciferase reporter assays. AA mouse xenograft tumor model was employed to investigate the function of circ-0002755 *in vivo*. They found that Sev suppressed viability, invasion, and migration and decreased lactate production and glucose consumption, while increasing apoptosis. Administration of Sev significantly reduced the expression of circ-0002755, while circ-0002755 was notably over-expressed in glioma tissues. They reported a three-way interaction between circ-0002755, mir628-5p, and MAGT1 and suggested that Sev could regulate the progression of glioma via the circ_0002755/miR-628-5p/MAGT1 axis. In addition, Sev suppressed the progression of glioma tumors *in vivo*.^367^

Recent studies have shown that circRNA Scm-like with 4 Mbt domain 2 (circ_SFMBT2) can act as a sponge of miR-182-5p, with a regulatory function in gastric adenocarcinoma growth.^368^ There is not much data about the putative downstream molecules of circ_SFMBT2 and the underlying mechanisms in glioma development, but the role of miR-182-5p in the pathogenesis of bladder and gastric cancer has been shown.^368^^,^^369^ miR-182-5p can control the growth of various kinds of tumors via metastasis suppressor 1 (MTSS1);^370^, ^371^, ^372^ MTSS1 has been correlated with tumor metastasis and progression in different cancers by complex interactions with the actin cytoskeleton.^371^^,^^372^ circ_SFMBT2 was shown to affect the growth of gastric cancer cells through targeting miR-182-5p.^368^

Zhang and colleagues investigated circ_SFMBT2 expression in glioma tissues.^373^ In their study, lower expression of circ_SFMBT2 was detected compared with normal astrocyte cells, and circ_SFMBT2 over-expression inhibited glioma cell growth *in vitro* and reduced metastasis. mir182-5p could be a downstream molecule of circ_SFMBT2, and they suggested that the circ_SFMBT2/mir-182-5p/MTSS1 axis could play a role in glioma treatment.^373^

circRNA homeodomain interacting protein kinase 3 (circHIPK3) and miR-124-3p were found to promote glioma development.^374^ In addition, miR-524-5p was found to regulate glioma cell proliferation.^375^ Kinesin family member 2A (KIF2A) plays an important role in cancer progression. Zhang et al. found that KIF2A could affect the prognosis of patients with nasopharyngeal carcinoma.^376^ KIF2A down-regulation inhibited gastric cancer invasion^377^ and could suppress oral SCC through inhibiting the PI3K/protein kinase B pathway.^378^ It was shown that KIF2A silencing could suppress migration and metastasis of glioma cells, while KIF2A knockdown could stimulate apoptosis *in vitro*.^379^

In a study by Yin et al., the effects of miR-524-5p, KIF2A, and circHIPK3 regulatory network on the development of TMZ-resistant glioma were investigated. This might shed light on the mechanisms involved in TMZ resistance and could improve TMZ treatment efficacy in glioma patients.^380^ qRT-PCR was used to measure serum levels of mRNAs, circRNAs, and miRNAs. The MTT assay was used to measure cell proliferation, and the TMZ half inhibitory concentration (IC_50_), western blotting, dual-luciferase reporter assay, flow cytometry, and transwell assays were employed to measure the level of protein expression, apoptosis, and metastasis, respectively. The results showed that circHIPK3 knockdown increased TMZ sensitivity in glioma and affected proliferation, apoptosis, and metastasis via the miR-524-5p/KIF2A-mediated PI3K/AKT pathway. This could be a new approach for diagnosis and therapy of TMZ-resistant glioma.^380^ Table 3 lists some metastasis-related circRNAs reported to be involved in GBM.

**Table 3:** Metastasis-related circular RNAs involved in GBM

circRNA	Expression status (up/down)	Target	Model (*in vitro*, *in vivo*, human)	Cell line	Ref
circCPA4	up	let-7	*in vitro*, *in vivo*, human	U25, U87	Peng et al.^114^
circ_SFMBT2	down	miR-182-5p	*in vitro*, human	D54, A172, U251	Zhang et al.^381^
circMMP9	up	miR-124	*in vitro*, *in vivo*, human	U87, U251, SHG44, A172, SNB19	Wang et al.^382^
circFBXW7	down	miR-23a-3p/PTEN	*in vitro*, *in vivo*, human	U251, U87, SHG44	Gao et al.^383^
circHIPK3	up	miR-124-3p	*in vitro*, human	T98G	Xia et al.^384^
circ_0002755	up	miR-628-5p/MAGT1	*in vitro*, *in vivo*, human	A-172, SHG-44	Li et al.^367^
circMMP1	up	miR-433/HMGB3	*in vitro*, *in vivo*, human	U251, LN229	Yin and Liu^385^
circTTBK2	up	miR-145-5p/CPEB4	*in vitro*, *in vivo*, human	T98G, LN229	Liu et al.^386^
hsa_circ_0030018	up	miR-1297/RAB21	*in vitro*, *in vivo*, human		Song et al.^387^
circHIPK3	up	miR-524-5p/KIF2A	*in vitro*, human	A172, U251	Yin and Cui^380^
circ_101064	up	miR-154-5p/PIWIL1	*in vitro*, human	U251, U87	Zhou et al.^125^
circ-U2AF1	up	hsa-miR-7-5p	*in vitro*, *in vivo*, human	U251, U87	Hamblin^388^
hsa_circ_0088732	up	miR-661/RAB3D	*in vitro*, *in vivo*, human	LN229, U251, A172 U87-MG	Jin et al.^389^
circPVT1	up	miR-199a-5p	*in vitro*, human	U539, U251	Chi et al.^390^
circSCAF11	up	miR-145-5p/miR-145-5p	*in vitro*, *in vivo*, human	T98G, LN229	Yin et al.^391^
hsa_circ_0067934	up	PI3K-AKT	*in vitro*, human	LN18, U251, LN229, T98G, A172	Xin et al.^392^
circ_0029426	up	miR-197	*in vitro*, human		Zhang et al.^393^

## Conclusion

It is now known that ncRNAs are involved in all phases of metastasis and can regulate invasion, migration, colony formation, and the development of the metastatic mass. ncRNAs may form complex interactions with other RNAs, DNA, and proteins to regulate the metastatic process. The exact signaling axes that are mediated by specific ncRNAs involved in the regulation of metastasis in individual cancer types should be elucidated by functional genomics assays. The latest advances in genome-editing techniques, such as CRISPR-Cas9, have made it possible to rapidly modify the human genome and could be used to study ncRNAs. Combining practical genetic screening with single-cell-based assays and confirming the results in animal models is the most attractive approach. These recent advances will shed more light on a thorough understanding of ncRNA function and the possible underlying molecular mechanisms involved in the regulation of metastasis. Clinical studies should be undertaken to verify the critical functions of ncRNAs in glioma development and metastasis. ncRNAs display a specific expression profile in many malignancies, which suggests they can serve as promising diagnostic and prognostic biomarkers in cancer as well as potential promising therapeutic targets for future cancer treatment.

ncRNAs could be a promising tool for targeted therapy combined with other new promising therapies. The development of a complete network of all ncRNAs involved in glioma formation and progression could supplement other therapeutic approaches (such as immunotherapy and gene therapy) and could also be used as a stratification tool for individualized treatment, resulting in an improved antitumor effect. The successful application of ncRNA-based therapeutics requires an unprecedented interdisciplinary approach, including technical advancements in molecular biology, immunology, pharmacology, chemistry, and nanotechnology. An optimal ncRNA therapeutic agent must be extensively tested for immunogenicity, chemically modified to improve its pharmacokinetics and pharmacodynamics, and delivered with consideration of its biodistribution and intracellular uptake mechanisms. It would need to specifically and potently interact with its intended target and be dosed at an appropriate level to trigger the desired effect. Studies in each of these areas should be carried out for each separate ncRNA therapeutic agent, but their successful translation will depend on further interdisciplinary collaboration to improve tolerance, specificity, and delivery.
